# Two Cases of Delerium Tremens Treated by the Tincture of Digitalis after Opium and Chloroform Had Failed

**Published:** 1866-12

**Authors:** 


*Two Cases of Delerium Tremens treated by the Tincture of Digitalis after Opium and Chloroform had failed.*

*Case* 1. Third day utter sleeplessness, with all the horrid symptoms of delerium tremens. Two teaspoonfuls of tincture of digitalis was ordered at once, and repeated in two hours. Sleep was procured in four and a half hours, but not till the pulse was made firmer.

*Case* 2. Was an old sot, who had suffered from repeated attacks of delerium tremens, which lasted longer at each recurrence. I was called to see him on the second day of the attack, and his brother told me that I had better kill him at once, as he was a miserable nuisance. I gave him f. ʒ vj., tincture of digitalis, as he was raving, violent, and almost unmanageable. In an hour he began to grow more manageable, and in three hours went into a profound sleep, which lasted three and a half hours, when he awoke nearly collected and much refreshed; f. ʒ ij. were then administered in ℥ vi. of beef tea, and the case was effectually cured in two days.

I was consulted concerning a delicate young lady, quite pale and anemic, æt. 23. The catamenia had never appeared. Hands and feet cold much of the time; shortness of breath; heart's murmur loud, hollow sound; palpitation; no desire to locomotion; occasional flushes of fever followed by headache; complains much of cardialgia and acid stomach; abdomen tympanitic; appetite morbid; bowels very irregular, sometimes quite costive, at others loose; has suffered from several attacks of hæmoptysis; sometimes the sclerotic coat of eye would be quite yellow, at others quite blue; tongue broad, partly white and pale, papilla very prominent; lungs sound; sleep quite imperfect, dreams very much; digestion quite in error. Has been treated by several physicians with iron, cod liver oil, and emmenagogues, all to no effect. Saw her six months ago; ordered ten drops of tincture digitalis three times a day. On the fifth day poisonous symptoms came on. Suspended the tincture for three days, and commenced with six drops three times a day. In ten days more toxic symptoms supervened again. Stopped for three days, when four drops of tincture was given three times a day, and continued thirteen days longer, when pain in pelvic cavity was felt, quite slightly at first, but increased in intensity for four days, when the flow came on quite lightly. The remedy was then given in two-drop doses three times a day, and by the forty-sixth day the flow was complete. Appetite gradually improved. The first notable symptom of improvement after the second toxic effect, was a feeling of agreeable warmth in the feet and hands, which was followed closely by more sound and refreshing sleep. The paleness of the mucous membrane of the lips, jaws and tongue began to pass away with the coldness of surface. She observed to me that her food gradually set lighter on her stomach, and that her heart symptoms were the first to feel improved.

This case greatly improved in sixty days, and cod liver oil was followed up for one hundred days in succession, and now she is in perfect health. Nothing but the tincture of digitalis was used for the first forty days. If we study this case closely, we shall find that the medicine in producing its toxic effect was followed by no permanent ill consequence; that its tonic power on the muscular fibre, under certain circumstances, is not to be disputed. What these circumstances are, constitutes the pith of the matter. I am of the opinion that the modus operandi is on the molecular life of the organ, throwing it into tonic movements. But I must not fail to show that the action is primarily on the heart, if not through the nutritive system. Digitalis has a power over the life forces that calls forth a greater power in the organs to select nutritive material from the blood. I must not be understood to say that I claim for it any power of enriching the blood. But we must not suppose that the blood is ever quite destitute of nutritive material. But when the vital forces are low, in consequence of imperfect nutrition, a general anemia of fibre occurs in consequence of imperfect supply of nutrition from impoverished blood.

But at the same time, if there exists a dormant life force in fibre or molecules, susceptible to stimulating action through the tonic power of digitalis, a renewal of life occurs, whose result is to call or select from the blood, poor as it may be at first, new material, which is followed by elimination of effete matter, and hence an awakening of life force through the whole nutritive system which calls for material to supply the waste, thereby making a demand for food. This is set forth in strong light by the fact that digitalis causes a large increase of solid excretia in the urine, without greatly increasing the quantity of water. This I repeatedly observed in pneumonia, for the demand for food was greater in cases treated with tincture of digitalis than when treated otherwise. It would be difficult to show that digitalis is a stimulant, that is in the usual acceptation of that term, nor can we call it a tonic in the sense that we do colombo and other tonics. However, we cannot fail to see that its effects are such as we should expect from a remedy that could appeal at once to the elementary molecules of tissue, and call or awaken them into renewed activity.

But its influence on the nervous system is manifestly different from that of narcotics proper, or of nervous sedatives. How it acts upon the excited brain and nervous system when suffering from alcoholism, can only be explained in the light of an antagonism to that poison on those tissues.—*Med. & Surg. Reporter.*

